# Guanidinylated SBA-15/Fe_3_O_4_ mesoporous nanocomposite as an efficient catalyst for the synthesis of pyranopyrazole derivatives

**DOI:** 10.1038/s41598-021-99120-3

**Published:** 2021-10-06

**Authors:** Fereshte Hassanzadeh-Afruzi, Somayeh Asgharnasl, Sara Mehraeen, Zeinab Amiri-Khamakani, Ali Maleki

**Affiliations:** Catalysts and Organic Synthesis Research Laboratory, Department of Chemistry, Iran University of Science and Technology, 16846-13114 Tehran, Iran

**Keywords:** Catalyst synthesis, Heterogeneous catalysis

## Abstract

In this study, a novel mesoporous nanocomposite was fabricated in several steps. In this regard, SBA-15 was prepared by the hydrothermal method, next it was magnetized by *in-situ* preparation of Fe_3_O_4_ MNPs. After that, the as-prepared SBA-15/Fe_3_O_4_ functionalized with 3-minopropyltriethoxysilane (APTES) via post-synthesis approach. Then, the guanidinylated SBA-15/Fe_3_O_4_ was obtained by nucleophilic addition of APTES@SBA-15/Fe_3_O_4_ to cyanimide. The prepared nanocomposite exhibited excellent catalytic activity in the synthesis of dihydropyrano[2,3-c]pyrazole derivatives which can be related to its physicochemical features such as strong basic sites (presented in guanidine group), Lewis acid site (presented in Fe_3_O_4_), high porous structure, and high surface area. The characterization of the prepared mesoporous nanocomposite was well accomplished by different techniques such as FT-IR, EDX, FESEM, TEM, VSM, TGA, XRD and BET. Furthermore, the magnetic catalyst was reused at least six consequent runs without considerable reduction in its catalytic activity.

## Introduction

**Figure 1:**
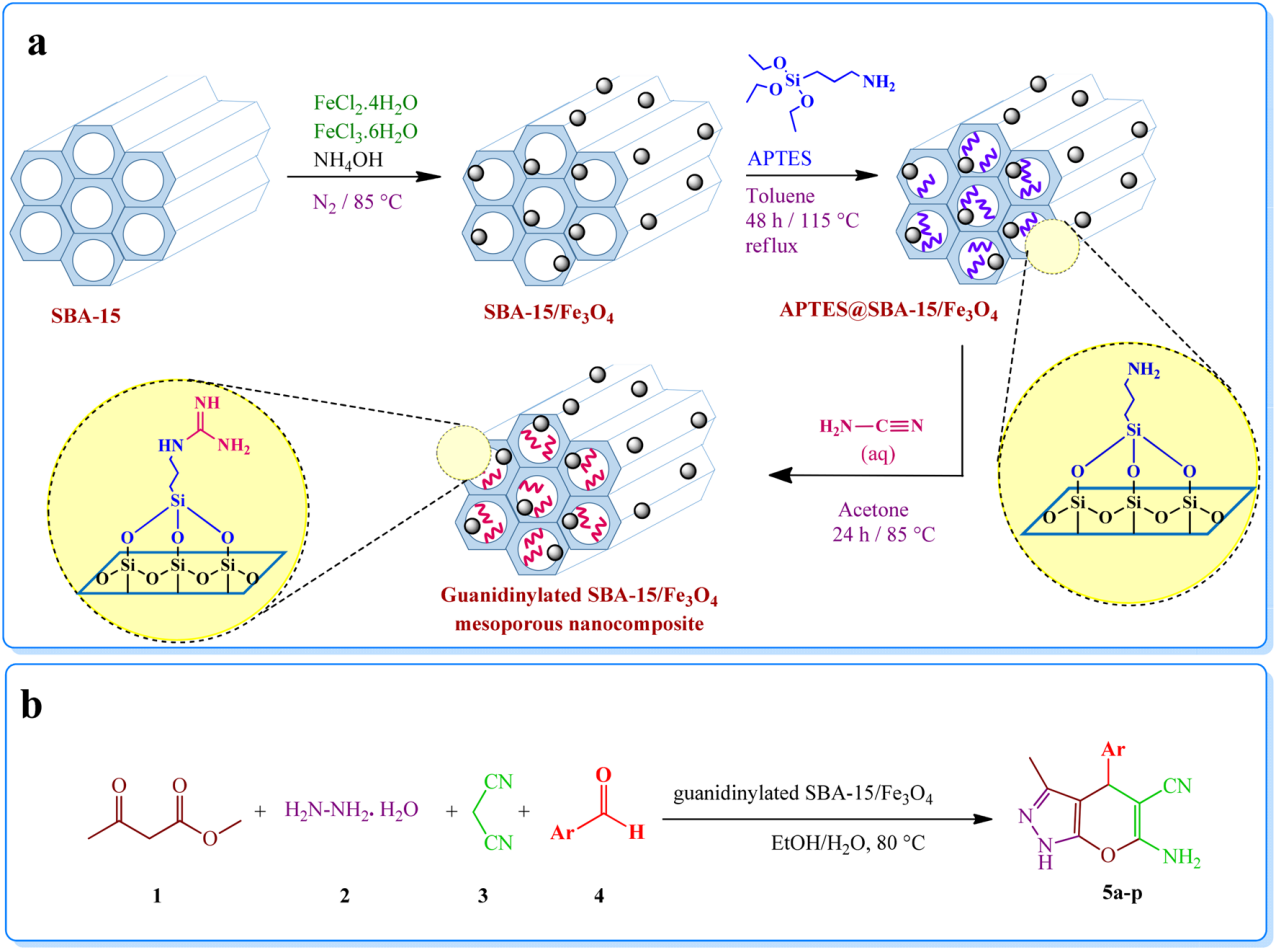
Preparation route of (**a**) the guanidinylated SBA-15/Fe_3_O_4_ and (**b**) its catalyst application in the synthesis of dihydropyrano[2,3-c]pyrazole derivatives.

Porous materials with remarkable characteristics such as high surface area, well-defined porous structure, uniform pore size, and processability have attracted great interest from researchers in scientific fields^1,2^. Based on the IUPAC definition, the porous materials are classified in three main groups depending on their pore size (or pore width): microporous (pores width < 2 nm), mesoporous (2 < pores width < 50 nm), and macroporous (pores width > 50 nm) materials^2,3^. In 1998, Stucky and et al. synthesized a novel kind of hexagonal array of pores named SBA (Santa Barbara Amorphous). After that, different types of SBA materials such as SBA-1, SBA-16 and SBA-15 have synthesized. The SBA-15 is a highly ordered mesoporous materials with striking properties for instance high surface area, straight cylindrical pores, thick framework walls, adjustable pore size (4–30), large pore diameter (which provide a facilitated diffusion of reactant molecules), and excellent ability to be modified/functionalized. It has been extensively utilized in various application such as catalysis^4^, removal of pollutants form wastewater^5^, hyperthermia^6^, drug delivery^7,8^, and chromatographic techniques^9^. To obtain high performance SBA-15-based catalysts, the surface functionalization or modification needs to be performed. There are two main approach to modify/functionalize the silica-based mesoporous materials including one-pot synthesis or co-condensation and grafting technique or post-synthesis. In the first approach, the active phase is added to the reaction mixture which subsequently co-assembles into the inorganic framework for the construction of the mesoporous material in single step, but in the second approach siliceous support is prepared followed by the modification with active moieties or their precursors^2,10,11^. Incorporating Fe_3_O_4_ MNPs as a superparamagnetic material into the silica-based mesoporous materials is a practical way to achieve retrievable and reusable nanocomposite with enhanced surface area^12^. The chemical modification of SBA-15 gives high efficiency porous catalyst by more active sites to interact with reactants. In recent years, a great attention has been dedicated to prepare amine functionalized SBA-15 to enhance its potential catalytic application. For instance, several types of amines functionalized SBA-15 were fabricated via grafting technique by using three different aminosilane reagents then, it was applied as heterogeneous catalysts for Michael addition^13^. One of the most widely used materials to modify the SBA-15 through both co-condensation or and post-synthesis is the 3-aminopropyltriethoxysilane (APTES)^14,15^. Guanidine, the nitrogenous analogue of carbonic acids, is a fascinating group of basic organic compounds. They are exceedingly strong Brønsted and Lewis bases, their basic strength more than amines, pyridines, diamines and amidines. This strong basicity is ascribed to great delocalization of positive charge on the guanidinium cation above the three nitrogen atoms. After protonation of guanidine group, the highly stable guanidinium ion act as a bidentate hydrogen bond donor which able to activate different hydrogen bond acceptors species such as carbonyl groups^16,17^. Therefore, guanidine and their derivatives can be considered as appropriate candidates in base-catalyzed organic reactions. The guanidinylation reaction has been used to convert primary amine groups in different materials for example chitosan^18,19^ and Poly(2-guanidinoethylmethacrylate)^20^ into guanidine groups. Multicomponent reactions (MCRs) are one of the most significant methods for the synthesis of heterocyclic compounds because of their outstanding properties such as high atomic economy, short reaction time, straightforward reaction model, high selectivity, and great compliance with principals of green chemistry^21–23^. Among the product of MCRs, the pyranopyrazole derivatives have received much interest of researchers due to their extensive application in pharmacology and medicine^24–27^. In continuation of research on heterogeneous nanocatalysts^28–30^, in this study a novel SBA-15 based nanocomposite prepared in four steps as illustrated in Fig. 1a, then it was used as a heterogeneous catalyst in the synthesis of dihydropyrano[2,3-c]pyrazole derivatives via four component condensation reaction (Fig. 1b).

## Experimental

### General

All the required chemical reagents and solvents were purchased from the chemical international companies including Merck and Sigma Aldrich. Several analyses were carried out to demonstrate the construction of the catalyst. Fourier-transform infrared (FT-IR) spectra were carried out by applying a Shimadzu IR-470 spectrometer by using KBr pellet. Elemental analysis of the prepared samples was performed by energy dispersive x-ray analysis (EDX) recorded on Numerix JEOL-JDX 8030 (30 kV, 20 mA). X-ray diffraction (XRD) pattern of fabricated samples were obtained by X-ray diffractometer (Bruker D8 Advance). The morphology and surface of samples were studied by a field emission scanning electron microscope (FESEM) and transmission electron microscopies (TEM) using VEGA2 TESCAN instrument and Zeiss-EM10C-100 kV, respectively. The magnetic behavior of samples was measured using VSM analysis (Meghnatis, daneshpajooh Kashan), and thermal stability of samples was studied by thermogravimetric analysis (TGA) using a BAHR-STA 504 instrument. Melting points of synthesized products were measured with an Electrothermal 9100 apparatus. The specific surface area of prepared samples was determined using the Brunauer–Emmett–Teller (BET, Micrometics ASAP2020). ^1^HNMR and ^13^C NMR nuclear magnetic resonance spectra were recorded on a Bruker DRX-500 spectrometer at 500 and 125 MHz, respectively. Analytical thin-layer chromatography (TLC) was done by Merck silica gel GF254 plates.

### Catalyst preparation

#### Preparation of SBA-15

According to the previously reported studies^31^, the SBA-15 was synthesized by the hydrothermal method. First, 3.5 g of P123 surfactant as a structure-directing agent was dissolved in 120 mL of 1.6 M hydrochloric acid. Next, the obtained solution stirred at 40 °C for 24 h to get a homogenous mixture. After that, 8 mL (7.5 g) of TEOS (tetraethyl orthosilicate) as an alkoxysilanes reagent was added into the above clear solution and stirring was continued at 40 °C for 24 h to complete hydrolysis of silica source. Then, the resulting mixture was transferred into the teflon-lined autoclave, and heated at 150 °C for 24 h. After the autoclave temperature gradually decreased, the resulting mixture was filtered, washed several consecutive times with distilled water (15 × 25 mL), and then dried at room temperature. The final white powder was calcined at 550 °C for 4 h with a heating rate of 2.3 °C min^-1^ for removal of surfactant and forming the ordered mesoporous channels.

#### Preparation of SBA-15/Fe_3_O_4_

To magnetize SBA-15, the *in-situ* co-precipitation method was performed. For this, 2 mmol (0.4 g) of FeCl_2_·4H_2_O and 4 mmol (0.54 g) of FeCl_3_.6H_2_O were dissolved in 50 ml distilled water. The obtained homogeneous solution was transferred to a 2-necked round-bottom flask containing 0.5 g dispersed SBA-15 in 50 mL distilled water. The final mixture was kept stirring at room temperature for about 45 min under N_2_ atmosphere. Then, the temperature was gradually raised to 85 °C. After that, 10 mL of ammonium hydroxide (25%) was added dropwise to the stirring solution. The reaction was kept under this condition for 2 h to form Fe_3_O_4_ MNPs on the mesoporous SBA-15 support. The synthesized magnetic product was collected by a magnet, washed with distilled water and acetone, and then dried in an oven at 90 °C for 5 h.

#### Preparation APTES functionalized SBA-15/Fe_3_O_4_ (APTES@SBA-15/Fe_3_O_4_)

The functionalization of SBA-15/Fe_3_O_4_ by APTES was carried out by the post-synthesis approach. At first, 0.5 g of SBA-15/Fe_3_O_4_ and 2 mL of APTES were dispersed in 35 mL of toluene by stirring. Then, the obtained mixture was refluxed at 115 °C for 48 h. It is expected that free silanol groups (Si–OH) of SBA-15/Fe_3_O_4_ covalently react with the silica group on the APTES to form Si–O-Si bond (siloxane bond) through condensation reaction. The dark brown precipitate was isolated by a magnet, washed several times with ethanol and acetone and then dried in oven at 100 °C.

#### Guanidinylation of APTES@SBA-15/Fe_3_O_4_

The guanidinylated SBA-15/Fe_3_O_4_ was obtained by nucleophilic addition between APTES @SBA-15/Fe_3_O_4_ and cyanamide. Initially, 0.5 g of cyanamide was dissolved in 5 ml distilled water and added to 0.1 g APTES@SBA-15/Fe_3_O_4_ which dispersed in 35 mL acetone; next the mixture was kept stirring for 24 h at 85 °C. Then, the final product collected by a magnet, washed several times with distilled water and acetone, and dried in oven at 100 °C.

### General procedure for the synthesis of pyranopyrazoles derivatives

The catalytic performance of fabricated mesoporous catalyst was evaluated in one-pot synthesis of the pyranopyrazoles. For this, four components including ethyl acetoacetate (1 mmol), hydrazine hydrate (1.2 mmol), aromatic aldehyde (1.0 mmol) and malononitrile (1.0 mmol) were mixed and reacted by adding 0.01 g of the guanidinylated SBA-15/Fe_3_O_4_ mesoporous catalyst in the presence of 1 ml EtOH/ H_2_O (1:1) at 80 °C. The reaction completion process was investigated by thin layer chromatography (TLC). After completion of the reaction, hot ethanol was added to dissolve the product, then undissolved magnetic mesoporous catalyst was separated from the reaction mixture by a magnet and filtration. The crude products were recrystallized from EtOH to obtain pure dihydropyrano[2,3-c] pyrazole derivatives.

### Spectral data of selected products

6-Amino-4-(4-chlorophenyl)-3-methyl-2,4-dihydropyrano[2,3-c]pyrazole-5-carbonitrile (**5b**): ^1^H-NMR (500 MHz, DMSO-d_6_): δ_H_ (ppm) = 1.79 (s, 3H, methyl), 4.63 (s, 1H, methine), 6.91 (s, 2H, NH_2_), 7.19–7.20 (d, J = 8 Hz, 2H, H-aromatic), 7.37–7.38 (d, J = 8 Hz, 2H, H-aromatic), 12.12 (s, 1H, NH); ^13^C NMR (125 MHz, DMSO-d_6_); δ_C_ (ppm) = 10.18, 36.04, 57.30, 97.66, 121.06, 128.91,129.82, 131.69, 136.12, 143.95, 155.19, 161.38.

6-Amino-3-methyl-4-(3-nitrophenyl)-1,4-dihydropyrano [2, 3-c] pyrazole-5-carbonitrile (**5e**): ^1^H-NMR (500 MHz, DMSO-d_6_): δ_H_ (ppm) = 1.81 (s, 3H, methyl), 4.88 (s, 1H, methine), 7.05 (s, 2H, NH_2_), 7.65 (m, 2H, H-aromatic), 8.02 (s, 1H, H-aromatic), 8.11 (dd, J = 7.7 Hz 1H, H-aromatic), 12.20 (s, 1H, NH); ^13^C NMR (125 MHz, DMSO-d_6_); δ_C_ (ppm) = 9.74, 35.66, 56.17, 96.65, 120.49, 121.83, 121.93, 130.22, 134.36, 135.89, 146.8, 147.88, 154.69, 161.13.

6-Amino-4-(4-hydroxyphenyl)-3-methyl-2,4-dihydropyrano[2,3-c]pyrazole-5-carbonitrile (**5k**): ^1^H-NMR (500 MHz, DMSO-d_6_): δ_H_ (ppm) = 1.78 (s, 3H, methyl), 4.47 (s, 1H, methine), 6.68–6.69 (d, J = 8 Hz, 2H, H-aromatic), 6.76 (s, 2H, NH_2_), 6.94–6.96 (d, J = 8 Hz, 2H, H-aromatic), 9.25 (s, 1H, OH) 12.03 (s, 1H, NH); ^13^C NMR (125 MHz, DMSO-d_6_); δ_C_ (ppm) = 10.2, 35.9, 58.3, 98.5, 115.6, 121.3, 128.9, 135.2, 135.9, 155.2, 156.5, 161.1.

## Results and discussion

### Characterizations

In the present work, a new magnetic mesoporous nanocomposite based on SBA-15 was prepared. In this regard, four main steps were carried out to obtain this catalyst include preparation of SBA-15, *in-situ* fabrication of Fe_3_O_4_ on SBA-15 support, functionalization of the SBA-15/Fe_3_O_4_ composite with APTES, and eventually the guanidinylation reaction of APTES@SBA-15/Fe_3_O_4_. To characterize the magnetic mesoporous catalyst, several spectral and analytical techniques were employed which will be explained and discussed.

#### FT-IR spectroscopy

**Figure 2:**
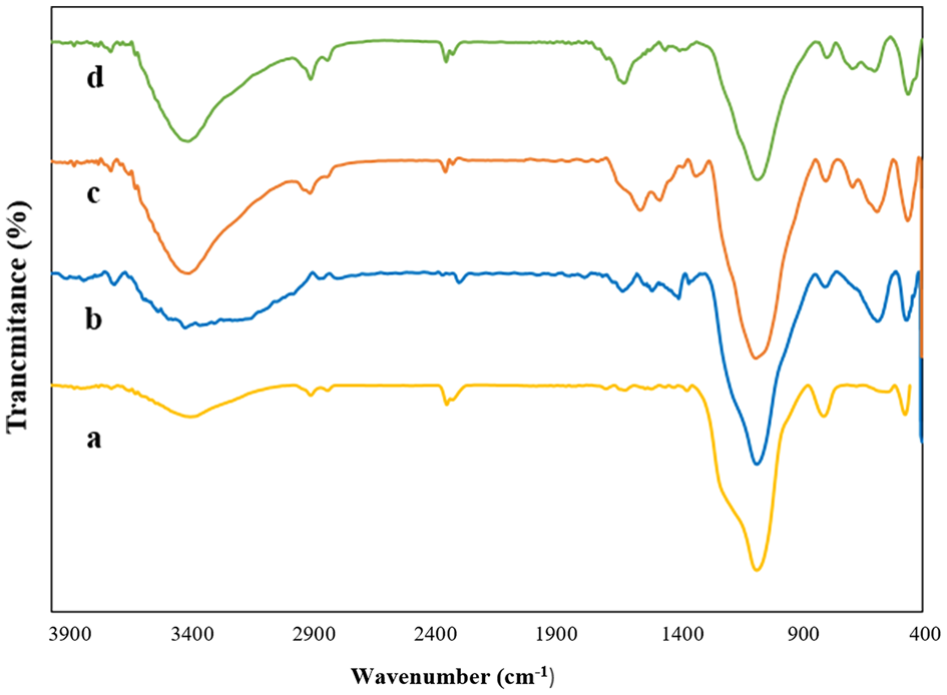
FT-IR of (**a**) SBA-15, (**b**) SBA-15/Fe_3_O_4_, (**c**) APTES@SBA-15/Fe_3_O_4_ and (**d**) guanidinylated SBA-15/Fe_3_O_4_.

FT-IR analysis was used to detect different functional groups in the samples fabricated in each step. As is observed in Fig. 2, IR spectrum of SBA-15 (a) has characteristic absorption bands at 470 (bending vibration of Si–O-Si), 800 (stretching vibration of Si–O-Si), 958 (vibration of Si–O-H), 1080 (symmetric stretching vibration of Si–O-Si), and 3420 cm^-1^ (stretching vibration of OH groups)^32–34^. IR spectrum of SBA-15/Fe_3_O_4_ in addition to similar absorptions which observed in spectrum a showed a new absorption bands at 577 cm^−1^ which assigned to Fe–O stretching vibration. After the functionalization of the SBA-15/Fe_3_O_4_ with APTES, two new absorption bands have emerged in 1562 and 2925 cm^-1^ which are related to the bending vibration of NH_2_ and the stretching vibrations of the C-H bond in the propyl chain of APTES, respectively (spectrum b) Furthermore, in this spectrum the intensity of absorption of the Si–OH bands at 958 cm^-1^ was decreased, signifying that the surface silanols are substituted by aminosilane groups after functionalization^35,36^. Moreover, guanidinylation of APTES @SBA-15/Fe_3_O_4_ was evaluated by comparing IR spectra of guanidinylated SBA-15/Fe_3_O_4_ (c) and APTES@SBA-15/Fe_3_O_4_ (b). Nucleophilic addition of APTES@SBA-15/Fe_3_O_4_ to cyanimide resulted in disappearing the bending vibration of NH_2_ at 1560 cm^-1^ and intensification the absorption band at 1627 cm^-1^ which can be attributed to the stretching vibrations of the imine bond of guanidine group formed during this final step modification^37^.

#### EDX analysis

**Figure 3:**
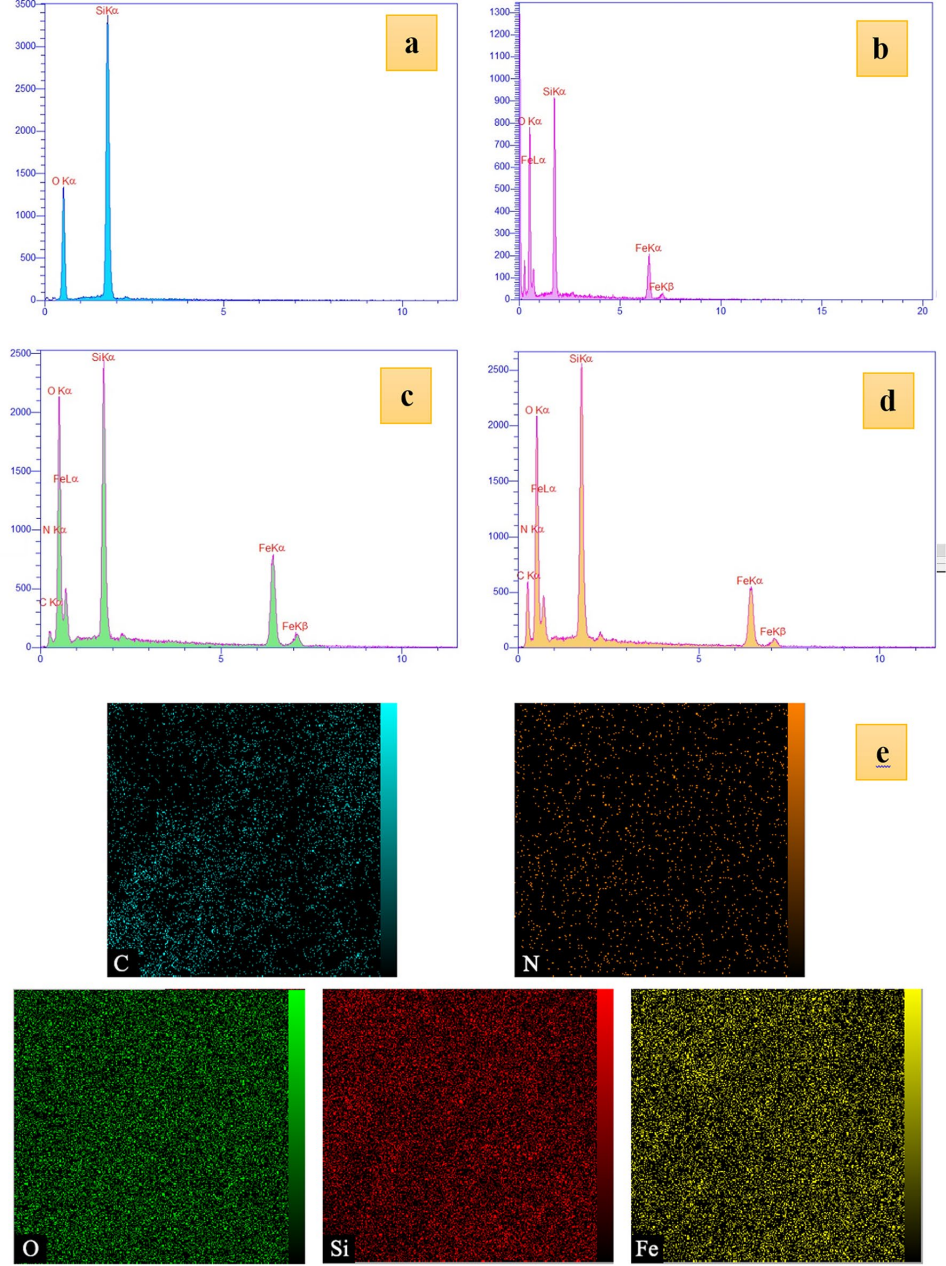
EDX analyses of (**a**) SBA-15**,** (**b**) SBA-15/Fe_3_O_4,_ (**c**) APTES@ SBA-15/Fe_3_O_4_, (**d**) guanidinylated SBA-15/Fe_3_O_4_, (**e**) and mapping images of the guanidinylated SBA-15/Fe_3_O_4_.

Detection of the organic and inorganic elements in the prepared samples was carried out by the EDX analysis as a qualitative method. As is shown in Fig. 3, O and Si are the elemental compositions of SBA-15(spectrum a), and distinctive peaks of Fe, O, and Si are related to the elemental composition of the SBA-15/Fe_3_O_4_ (spectrum b), observing _two_ Fe peaks are related to the existence of Fe_3_O_4_ MNPs in the structure of the SBA-15/Fe_3_O_4_. By modification of the SBA-15/Fe_3_O_4_ with APTES, the peaks of C and N elements were added to the previous peaks in the spectrum c. The guanidinylated SBA-15/Fe_3_O_4_ can cause the emerging of Fe, O, Si, C and N peaks in the EDX spectrum d. Moreover, the distribution of elements in this mesoporous nanocomposite is shown in the EDX mapping images (e).

#### Transmission and Scanning Electron Microscopies (TEM, SEM)

**Figure 4:**
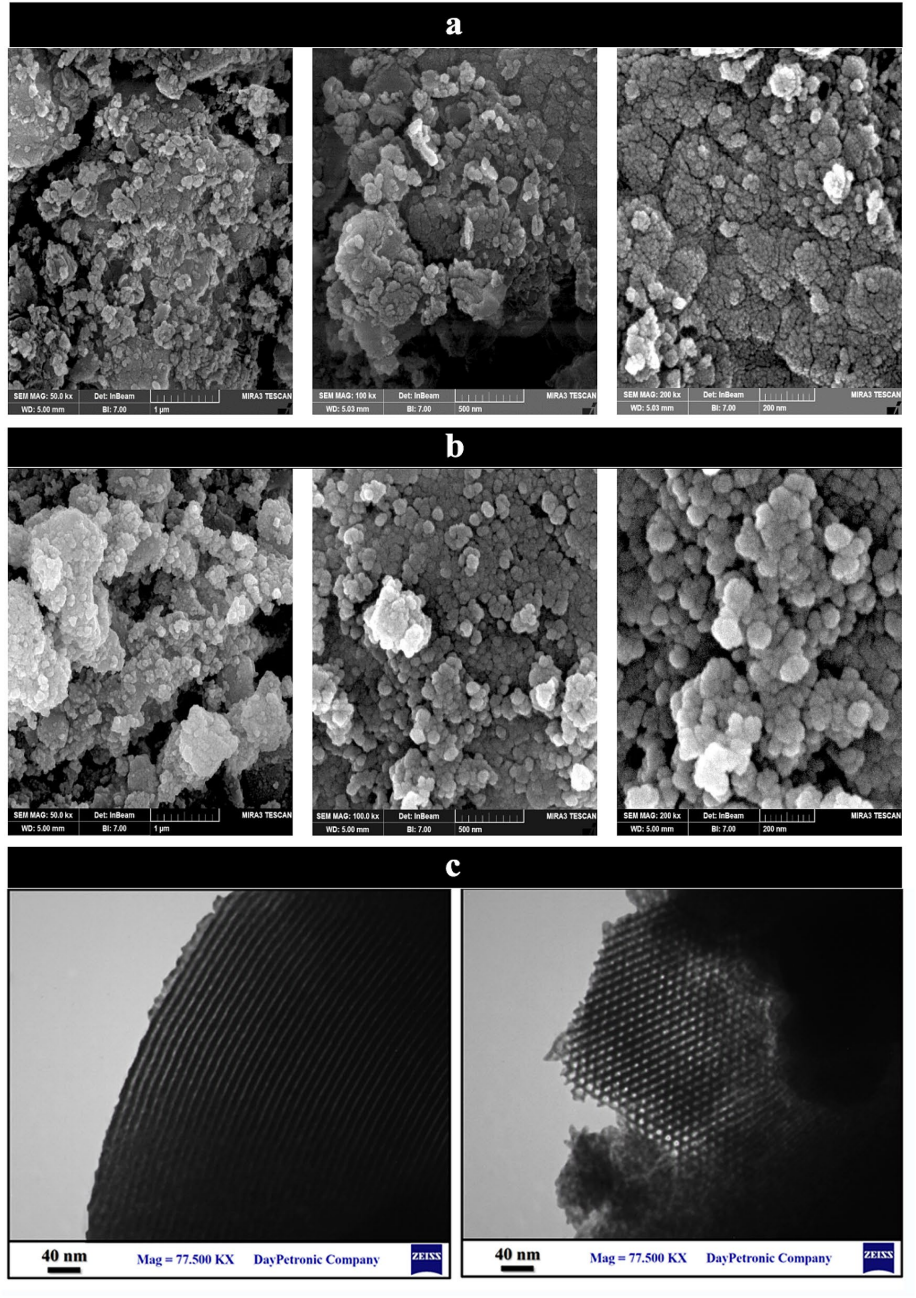
FESEM images of (**a**) SBA-15, (**b**) the guanidinylated SBA-15/Fe_3_O_4_, and (**c**) TEM image of the guanidinylated SBA-15/Fe_3_O_4_.

**Figure 5:**
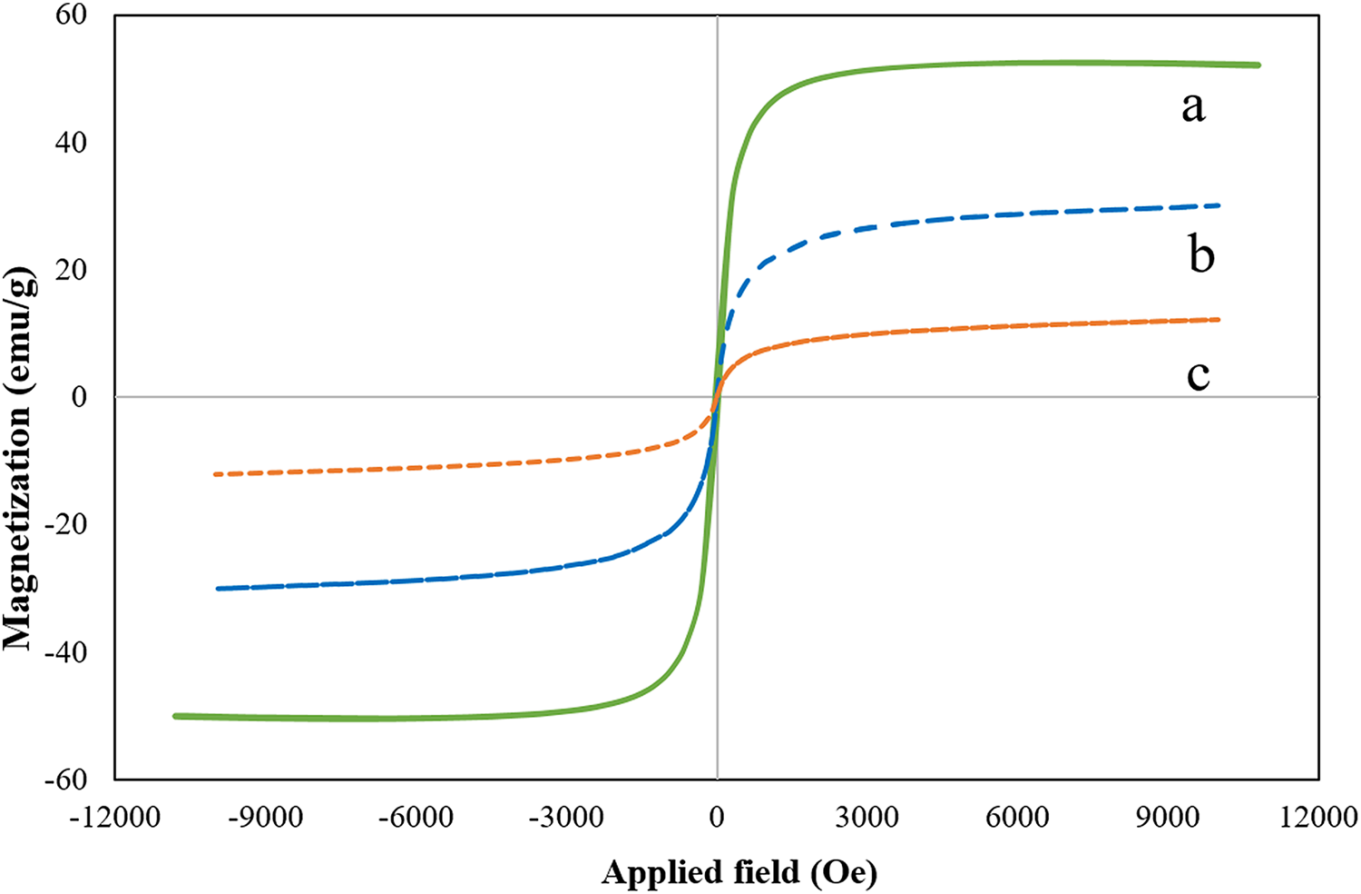
The magnetization curves of (**a**) Fe_3_O_4_ MNPs, (**b**) SBA-15/Fe_3_O_4_, (**c**) and the guanidinylated SBA-15/Fe_3_O_4_.

Field emission scanning electron microscopy was employed to observe particle size distribution, surface morphology and particle aggregation mode in prepared samples. As is observed in Fig. 4, the FESEM images of guanidinylated SBA-15/Fe_3_O_4_ and SBA-15 are presented in three scales: 1 µm, 500 and 200 nm. The SBA-15 images presented a porous structure, but in the nanocomposite images in addition to the porous structure, the distribution of spherical Fe_3_O_4_ MNPs on the SBA-15 as a mesoporous support was also can be observed. Therefore, fabrication Fe_3_O_4_ MNPs onto SBA-15 mesoporous matrix and subsequent modification resulted in change the its morphology. The average particle size for 35 spherical particles in the nanocomposite was determined to be about 26 nm using Digimizer software. TEM analysis was performed to more accurately study the morphology and particle size of the mesostructured guanidinylated SBA-15/Fe_3_O_4_ catalyst. As can be seen in Fig. 5, TEM image of prepared nanocomposite (left image of Fig. 4c) exhibited an ordered pore channels framework having dimension about 6–7 nm. In another image (right image of Fig. 4c), both a regular mesoporous arrangement with two-dimensional hexagonal honeycomb structure and the Fe_3_O_4_ MNPs onto SBA-15 support was observed, but formation of the magnetite NPs onto the certain amount of SBA-15 channels and subsequent functionalization lead to hide some part of ordered pore arrangement.

#### VSM analysis

To evaluate the magnetic properties of the prepared samples, vibrating sample magnetometer (VSM) was employed at room temperature and in the field range of -10 < kOe <  + 10. As indicated in Fig. 5, the measured magnetic saturation (Ms) for bare Fe_3_O_4_, SBA-15/Fe_3_O_4_ and guanidinylated SBA-15/Fe_3_O_4_ were about 52.1, 30.1, and 12.16 emu/g, respectively. The reduction in Ms of the SBA-15/Fe_3_O_4_ compared to bare Fe_3_O_4_ is due to the presence of SBA-15 as a non-magnetic component in the SBA-15/Fe_3_O_4_ structure. Then, by further chemical modification on SBA-15/Fe_3_O_4_ and the addition of more non-magnetic segments, the amount of Ms in guanidinylated SBA-15/Fe_3_O_4_ was decreased to about 12.16 (emu/g), which seems reasonable. However, the nanocomposite is sufficiently magnetized to be effortlessly separated from the reaction mixture by a magnet and reused for several consecutive times. Besides, considering that the values of coercivity (Hc) and remanence (Mr) in the VSM curves of the prepared samples are zero, their superparamagnetic behavior is confirmed.

#### TGA analysis

**Figure 6:**
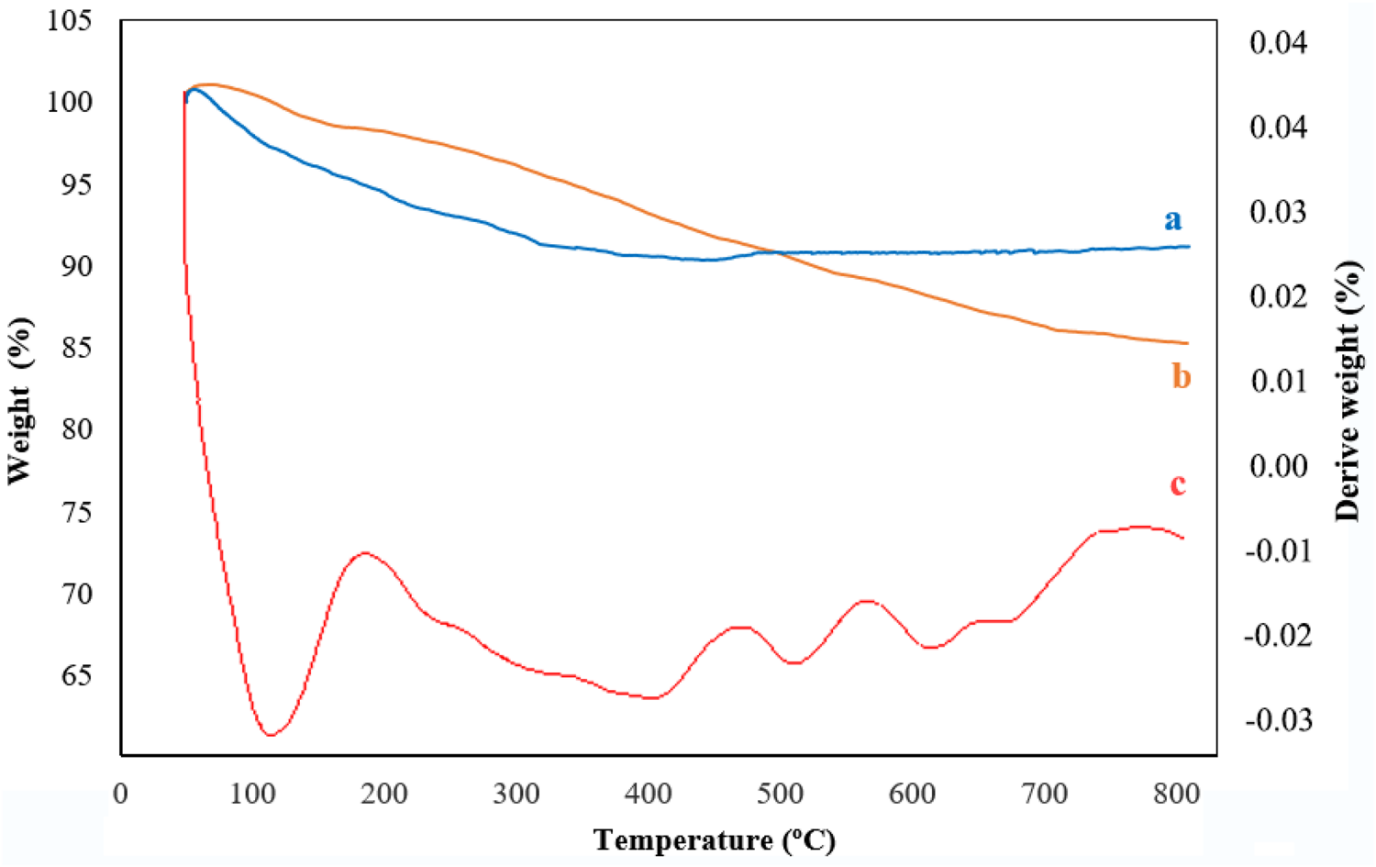
TGA curves of (**a**) SBA-15/Fe_3_O_4_, (**b**) the guanidinylated SBA-15/Fe_3_O_4_, and (**c**) DTGA of the guanidinylated SBA-15/Fe_3_O_4_.

Thermal analysis of the SBA-15/Fe_3_O_4_ and guanidinylated SBA-15/Fe_3_O_4_ was performed by TGA analysis in the temperature range 50–800° C with a heating rate of 10 ºC min^-1^ under air atmosphere. For a more detailed study, the thermal behavior of SBA-15 and Fe_3_O_4_ which reported in literature was also studied. According to the reported information, SBA-15 has high thermal stability; it has maintained above 90–95% of its weight up to 700 ºC and a continuous slight weight loss was attributed to dehydrogenation or dehydroxylation of its surface^38,39^. Moreover, the thermal behavior of Fe_3_O_4_ displayed that with increasing the temperature to 800 °C, a small weight loss of about 5–6% was occurred, which was ascribed to the evaporation of water molecules absorbed in it^40^. As can be observed in Fig. 6, the thermogram of the SBA-15/Fe_3_O_4_ (a) exhibited a gradual gentle weight loss between 50 and 800° C which may be related to the evaporation of adsorbed water molecules in the cavities of this sample, and dehydrogenation or dehydroxylation of its surface. Therefore, high thermal stability of this composite was demonstrated with just 9% weight loss until 800 °C. In the thermogram of guanidinylated SBA-15/Fe_3_O_4_ nanocomposite (b), the first observed weight loss (~ 2%) in the temperature range of 50–160 °C is attributed to the evaporation adsorbed water molecules in the cavities and surface of the mesoporous nanocomposite. Next, with increasing temperature to 550 °C weight loss of about 10% occurs, which can be due to the separation and thermal decomposition of organic parts (alkyl chain and guanidine group) that covalently bounded to the SBA-15/Fe_3_O_4_, and the continuation of weight loss with increasing temperature up to 800 °C can be ascribed to the condensation of silanol groups of guanidinylated SBA-15/Fe_3_O_4_^41^. The residual weight of these mesoporous nanocomposites up to 800 °C is about 85%, which is only 6% more weight loss than the SBA-15/Fe_3_O_4_ compound. Therefore, it can be referred that the two steps chemical modification of SBA-15/Fe_3_O_4_ did not have a significant effect on its thermal resistance. Furthermore, considering the difference in residual weight of the two samples, it can be calculated that just about 6% of the total weight of the guanidinylated SBA-15/Fe_3_O_4_ nanocomposite composed of the organic part. The differential thermogravimetric analysis (DTGA) of guanidinylated SBA-15/Fe_3_O_4_ (spectrum c) showed main endothermic peaks at 123 and two weak peaks at 411 and 510, the first one is attributed to the loss of adsorbed water molecules in mesoporous nanocomposite and the others related to decomposition of the organic part which constitute a very small weight percent of nanocomposite and the condensation of silanol groups of SBA-15. It can be seen from these results that the prepared mesoporous nanocomposite has structural stability at high temperatures and can be used for catalytic reactions at high temperature.

#### XRD analysis

**Figure 7:**
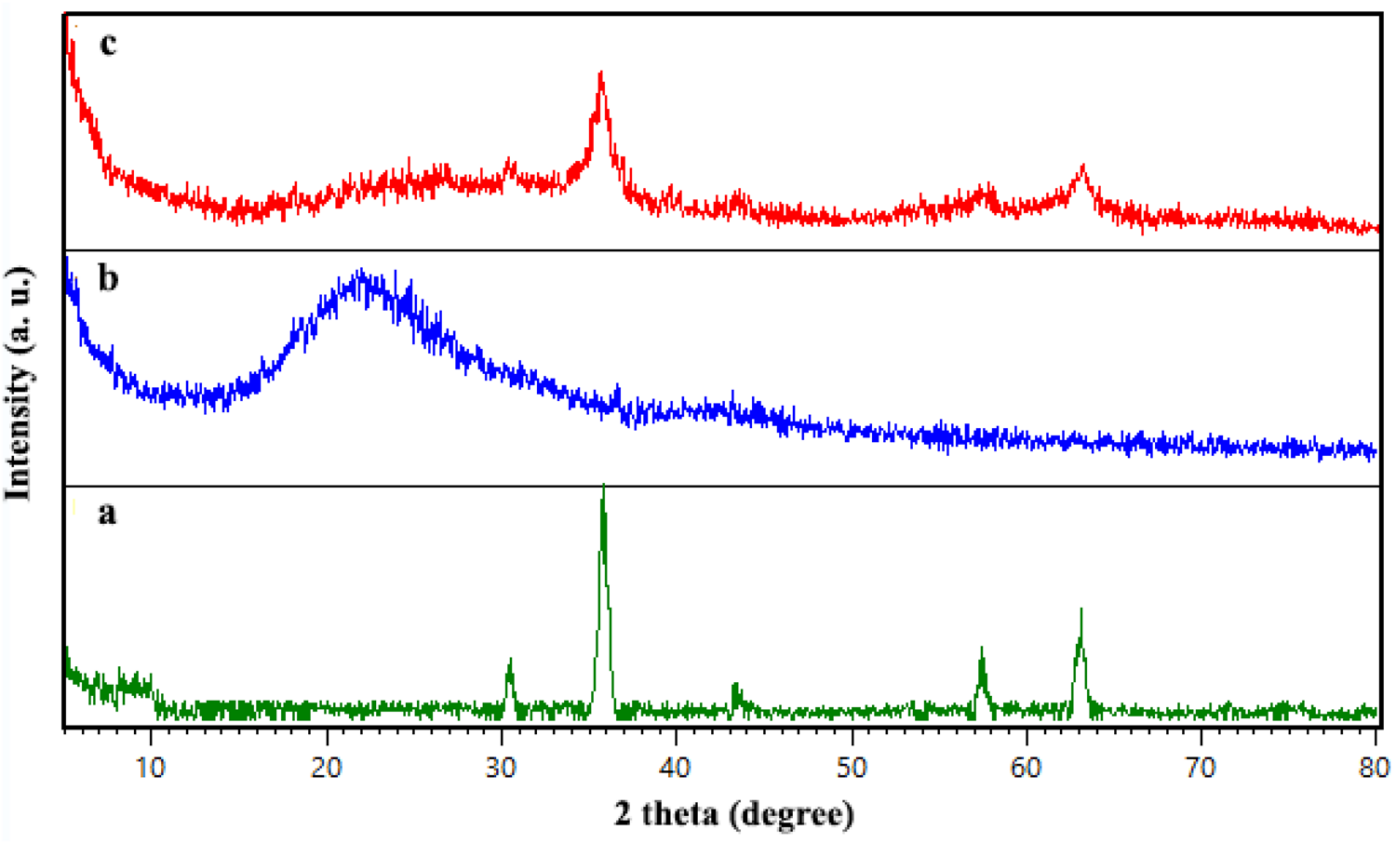
XRD pattern of (**a**) Fe_3_O_4_, (**b**) SBA-15 and (**c**), the guanidinylated SBA-15/Fe_3_O_4_.

**Figure 8:**
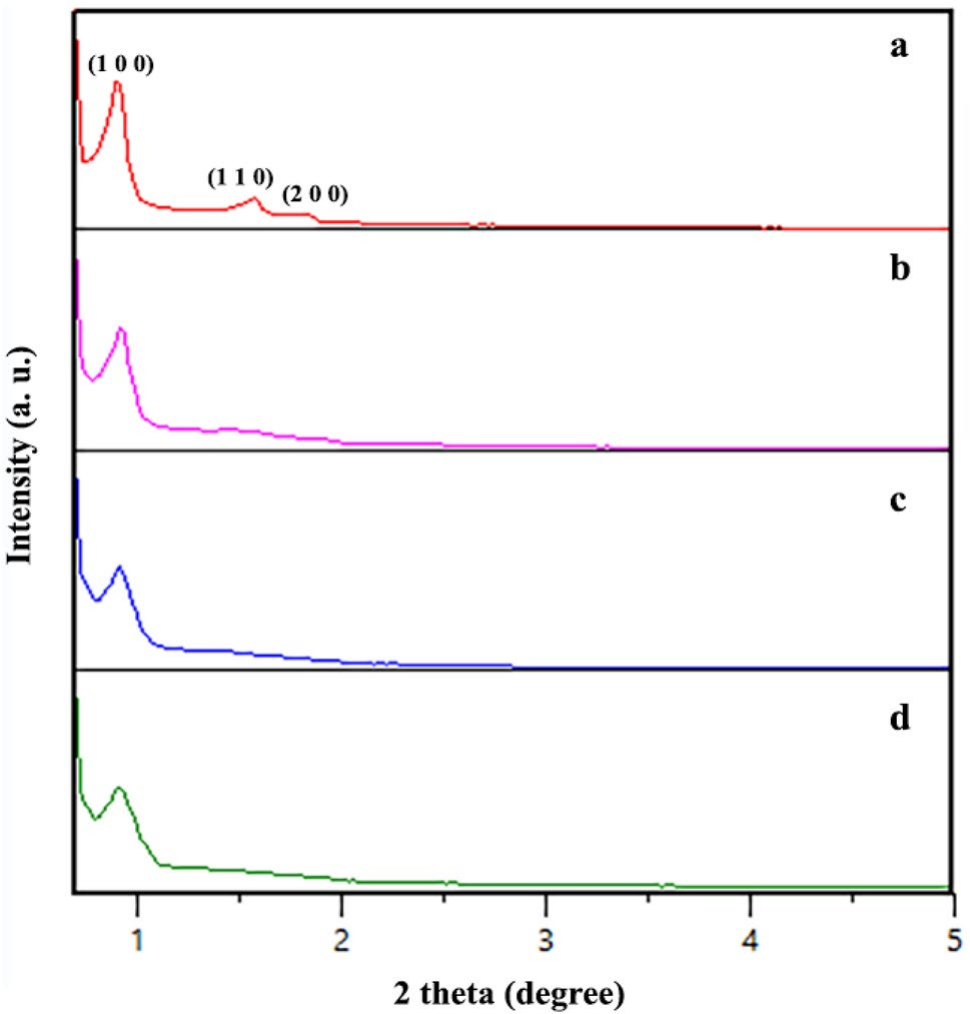
Low-angle XRD patterns of (**a**) SBA-15, (**b**) SBA-15/Fe_3_O_4_, (**c**) APTES@SBA-15/Fe3O4, and (**d**) the guanidinylated SBA-15/Fe_3_O_4._

The XRD analysis was used to investigate the crystalline nature of prepared samples. As is depicted in Fig. 7, the XRD pattern of SBA-15 (b) showed a broad characteristic peak at 2Ө: 20–30^34,42^.The diffractogram of guanidinylated SBA-15/Fe_3_O_4_ (c) exhibited the relatively broad peak at 2Ө: 20–30° with attributed to the presence of SBA-15, which was lower intensity compared to unmodified SBA-15, this reduction can be ascribed to three steps of modification of SBA-15. Moreover, there are several peaks at 2Ө: 30.38°, 35.74°, 43.40°, 58°, 63.22° correspond exactly to the index peaks in the XRD pattern of Fe_3_O_4_ confirming the presence of these nanoparticles in the guanidinylated SBA-15/Fe_3_O_4_ structure. It can be concluded that formation of Fe_3_O_4_ MNPs on SBA-15 matrix even with modifications has enhanced its crystallinity. The average crystallite size of neat Fe_3_O_4_ and the guanidinylated SBA-15/Fe_3_O_4_ nanocomposite by the Scherer equation was calculated to be about 19 and 37 nm, respectively. The increase in average crystallite size of mesoporous nanocomposite in compared with neat Fe_3_O_4_ can be mainly due to the SBA-15 matrix Furthermore, the X-ray diffraction patterns of all prepared samples at low angles in the 2Ө: 0–5° are shown in Fig. 8. The XRD pattern of SBA-15 shows one high intensity peak at 2Ө: 0.91 and two small peaks at 2Ө: 1.58 and 1.82 corresponding to (1 0 0), (1 1 0) and (2 0 0) planes, respectively. They are typical hexagonally structured SBA-15 with highly ordered mesoporous channels. As observed, diffractograms of SBA-15/Fe_3_O_4_, APTES@SBA-15/Fe_3_O_4_ and guanidinylated SBA-15/Fe_3_O_4_ samples showed the first peak corresponding to the (100) reflection which intensity decreases with each step modification, but two minor peaks corresponding to (1 1 0) and (2 0 0) planes are became weaker or almost disappeared in these samples which can be attributed to partial disruption of the structure by incorporation of Fe_3_O_4_ MNPs and the lowering of the local order.

#### The N_2_ adsorption–desorption isotherm

**Figure 9:**
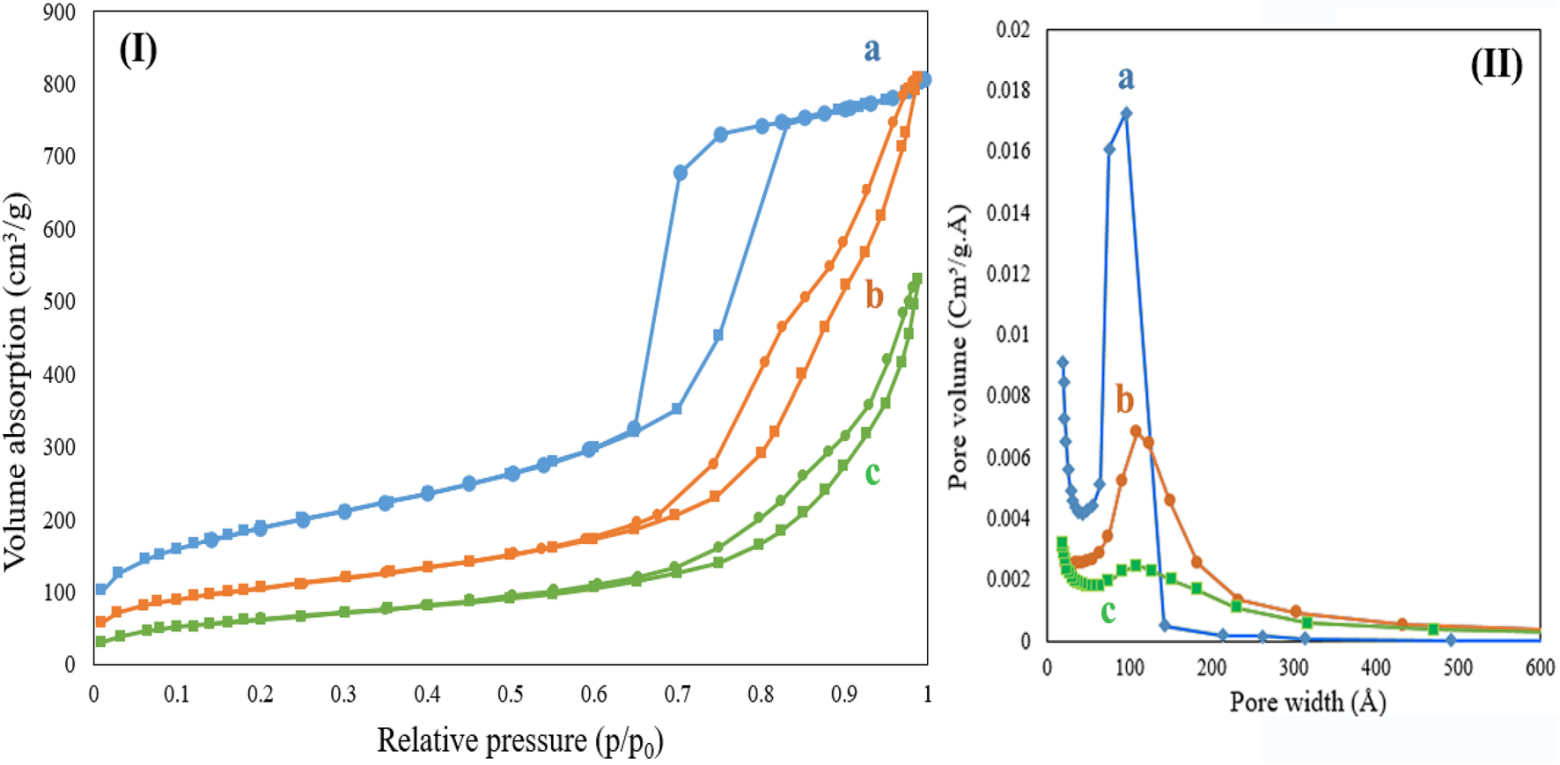
(**I**) N_2_ adsorption–desorption isotherms and (**II**) the pore size distribution curve of (**a**) SBA-15, (**b**) SBA-15/Fe_3_O_4_ and (**c**), the guanidinylated SBA-15/Fe_3_O_4_.

The N_2_ adsorption–desorption isotherm of the SBA-15, SBA-15/Fe_3_O_4_ and guanidinylated SBA-15/Fe_3_O_4_ are presented in Fig. 9. All samples exhibited a typical type-IV curves which are characteristic of mesoporous materials. However, the magnetic nanocomposites exhibited narrower hysteresis cycles than the SBA-15 and their adsorption and desorption branches are closer which can be related to an increase in their pore diameters, as can be seen in Fig. 9(I). The surface area, the pore volume and pore size (width) were calculated by the BET (Brunauer–Emmett–Teller) and BJH (Barrett–Joyner–Halenda) methods and results are summarized in Table 1. The BET surface area of the bare SBA-15 is about 687 m^2^/g which is more than the BET surface area of the SBA-15/Fe_3_O_4_ and guanidinylated SBA-15/Fe_3_O_4_. This observation can be mainly related to blocking of a certain amount of SBA-15 pores by the embedding of Fe3O4 MNPs on the SBA-15 pores and its subsequent functionalization, which resulted in less available surface area for gas adsorption. BJH pore size distributions of samples are depicted in Fig. 9(II), it is observed that the SBA-15/Fe_3_O_4_ and guanidinylated SBA-15/Fe_3_O_4_ samples has slightly more pore width (size) and also relatively broader pore size distributions compared to their precursor matrix.

**Table 1:** Surface area, pore volume and pore diameter of SBA-15/Fe_3_O_4_ and guanidinylated SBA-15/Fe_3_O_4_ nanocomposite.

Sample	Surface area^a^ (m^2^/g)	Pore volume^b^ (cm^3^/g)	Pore size^b^ (nm)
SBA-15	686.90	1.28	7.35
SBA-15/Fe_3_O_4_	383.91	1.26	12.64
guanidinylated SBA-15/Fe_3_O_4_	232.11	0.83	13.30

^a^The surface area obtained by BET analysis.

^b^Pore volume and pore diameter obtained by BJH analysis.

### The catalytic application of guanidinylated SBA-15/Fe_3_O_4_ nanocomposite

The catalytic activity of the guanidinylated SBA-15/Fe_3_O_4_ investigated in the synthesis of dihydropyrano [2, 3-c] pyrazole derivatives. To obtain the best result, different experimental conditions such as temperature, solvent, amount of catalyst and the type of catalysts was examined in the one-pot four components reaction of ethyl acetoacetate (1 mmol), hydrazine hydrate (1.2 mmol), 3-nitrobezaldehyde (1 mmol) and malononitrile (1 mmol). First, the model reaction was performed without catalyst and solvent in two different temperatures, the yield of products was trace (Table 2, entries 1 and 2). By adding the guanidinylated SBA-15/Fe_3_O_4_ catalyst to the model reaction in the absence of solvent, the yield of the reaction was reached about 47% (Table 2, entry 3). In the next step, to assess the solvent effect, ethanol was added to the reaction in the presence of a catalyst at room temperature, the efficiency increased considerably (Table 2, entry 4). Then, the reaction was repeated at 80° C to evaluate the effect of temperature and observed that increasing the temperature up to 80° C leads to the reaction progress (Table 2, entry 5). The Subsequent optimization experiments were performed in H_2_O and EtOH/H_2_O as green media under reflux and ultrasonic conditions; the best efficiency was obtained in EtOH/H_2_O media at 80 °C (Table 2, entries 6–8). In addition to reaction condition and solvent, various amounts of mesoporous nanocatalyst were tested and the maximum yield of product was achieved in the presence of 0.015 g catalyst (Table 2, entries 9 and 10). The efficiency of the prepared mesoporous nanocomposite compared with Fe_3_O_4_, SBA-15/Fe_3_O_4_, and APTES@SBA-15/Fe_3_O_4_ in the model reaction was examined. As indicated in Table 2 (entries 11–13), the yield of a reaction in the presence of guanidinylated SBA-15/Fe_3_O_4_ is higher than APTES@SBA-15/Fe_3_O_4,_ SBA-15/Fe_3_O_4_, Fe_3_O_4_, respectively. Indeed, guanidinylated SBA-15/Fe_3_O_4_ mesoporous nanocatalyst with dual active sites: base (primary and secondary amines), Lewis acid site (Fe^3+^ in Fe_3_O_4_), high porosity and large surface area acted as an excellent catalyst to accelerate the synthesis of dihydropyrano [2,3-c] pyrazole derivatives via one-pot four-component reaction.

**Table 2:** Optimizing the reaction conditions in the synthesis of dihydropyrano[2,3-c]pyrazole derivatives.

Entry	Catalyst	Catalyst loading (g)	Solvent	Temp (ºC)	Yield^a^ (%)
1	**–**		**–**	r.t	**Trace**
2	**–**		**–**	80	**Trace**
3	guanidinylated SBA-15/Fe_3_O_4_	0.02	**–**	r.t	**47**
4	guanidinylated SBA-15/Fe_3_O_4_	0.02	EtOH	r.t	**70**
5	guanidinylated SBA-15/Fe_3_O_4_	0.02	EtOH	80	**85**
6	guanidinylated SBA-15/Fe_3_O_4_	0.02	H_2_O	100	** < 60**
7	guanidinylated SBA-15/Fe_3_O_4_	0.02	EtOH/H_2_O (1:1)	80	**95**
8	guanidinylated SBA-15/Fe_3_O_4_	0.02	EtOH/H_2_O (1:1)	Ultrasonic, r.t	**75**
9	guanidinylated SBA-15/Fe_3_O_4_	0.01	EtOH/H_2_O (1:1)	80	**76**
**10**	**guanidinylated SBA15/Fe**_**3**_**O**_**4**_	**0.015**	**EtOH/H**_**2**_**O (1:1)**	**80**	**95**
11	Fe_3_O_4_	0.015	EtOH/H_2_O (1:1)	80	**N.R**
12	SBA-15/Fe_3_O_4_	0.015	EtOH/H_2_O (1:1)	80	**71**
13	APTES @SBA-15/Fe_3_O_4_	0.015	EtOH/H_2_O (1:1)	80	**84**

^a^Reaction conditions: ethyl acetoacetate (1 mmol), hydrazine hydrate (1.2 mmol), 3-nitrobezaldehyde (1 mmol) and malononitrile (1 mmol), catalyst (10–20 mg).

^b^The yields relate to the isolated product.

To assess the generality of the optimum conditions, a wide range of benzaldehydes bearing both electron-donating and electron-withdrawing substitution were tested. As is observed in Table 3, a wide range of substituted dihydropyrano[2,3-c]pyrazole derivatives were obtained in high yields by using guanidinylated SBA-15/Fe_3_O_4_ mesoporous nanocatalyst in short reaction times.

**Table 3:** The synthesis of dihydropyrano[2,3-c] pyrazole derivatives in optimized condition using the guanidinylated SBA-15/Fe_3_O_4_.


Entry	R1	Product	Time (min)	Yield^a^ (%)	Mp (ºC)	
					Observed	Literature
1	2-Cl	**5a**	25	91	238–241	241–244^33^
2	4-Cl	**5b**	20	92	230–232	232–233^24^
3	2,4-Cl_2_	**5c**	30	90	196–198	198–199^25^
4	2-NO_2_	**5d**	25	92	225–228	227–228^25^
5	3-NO_2_	**5e**	20	95	196–198	195–196^34^
6	4-Me	**5f.**	20	93	202–204	204–206^33^
7	3-OMe	**5g**	20	92	243–245	244–245^43^
8	4-OMe	**5h**	30	92	208–210	210–212^24^
9	3,4,5-(OMe)_3_	**5i**	40	89	208–211	210–212^24^
10	3-OH	**5j**	30	89	255–257	253–256^35^
11	4-OH	**5k**	25	90	223–225	223–225^24^
12	3,4-(OH)_2_	**5l**	40	89	175–177	175–178^26^
13	4-F	**5m**	20	96	203–205	205–207^26^
14	4-CH(Me)_2_	**5n**	20	90	213–215	211–213^25^
15	4-Br	**5o**	30	90	239–242	242–246^35^
16	2-OH-5-Br	**5p**	30	95	226–228	226–227^24^

^a^The yields relate to the isolated product.

To assess the catalytic efficiency and advantages of the guanidinylated SBA-15/Fe_3_O_4_ nanocomposite as an appropriate catalyst to promote the dihydropyrano[2,3-c], its catalytic activity in the synthesis of **5e** derivatives was compared with some other previously reported methods. As indicated in Table 4, the present method is superior to the other methods in terms of the yields of products or the reaction condition.

**Table 4:** Comparison of the catalytic performance of the guanidinylated SBA-15/Fe_3_O_4_ with some other reported catalysts for the synthesis of dihydropyrano[2,3-c]pyrazole (product **5e**).

Entry	Catalyst	Catalyst loading	Conditions	Yield^a^ (%)	References
1	Lemon peel powder	10 wt%	EtOH, reflux	74	^44^
2	cetyltrimethylammonium chloride(CTACl)	20 mol%	H_2_O, 90 °C	90	^45^
3	OPC-SO_3_H	0.02g	EtOH, 80 °C	89	^46^
4	Molecular sieves (MS 4 Å)	10 ml	EtOH, reflux	84	^47^
5	Sodium ascorbate	15 mol%	H_2_O, reflux	84	^48^
6	BF_3_/MNPs	0.1 g	EtOH, 80 °C	91	^49^
7	TEA-Im-IL-Cu	200 ml	H_2_O, 80 °C	85	^50^
8	L-proline	10 mol%	H_2_O, reflux	87	^51^
9	nano-Al_2_O_3_/BF_3_/Fe_3_O_4_	0.03 g	EtOH / H_2_O, reflux	85	^52^
10	γ-alumina	30 mol%	H_2_O, reflux	75	^53^
11	Aspartic acid	20 mol%	EtOH / H_2_O, r.t	84	^54^
**12**	**guanidinylated SBA-15/Fe**_**3**_**O**_**4**_	**0.03 g**	**EtOH / H**_**2**_**O, 80 °C**	95	**This work**

^a^The yields relate to the isolated product.

### Suggested mechanism

**Figure 10:**
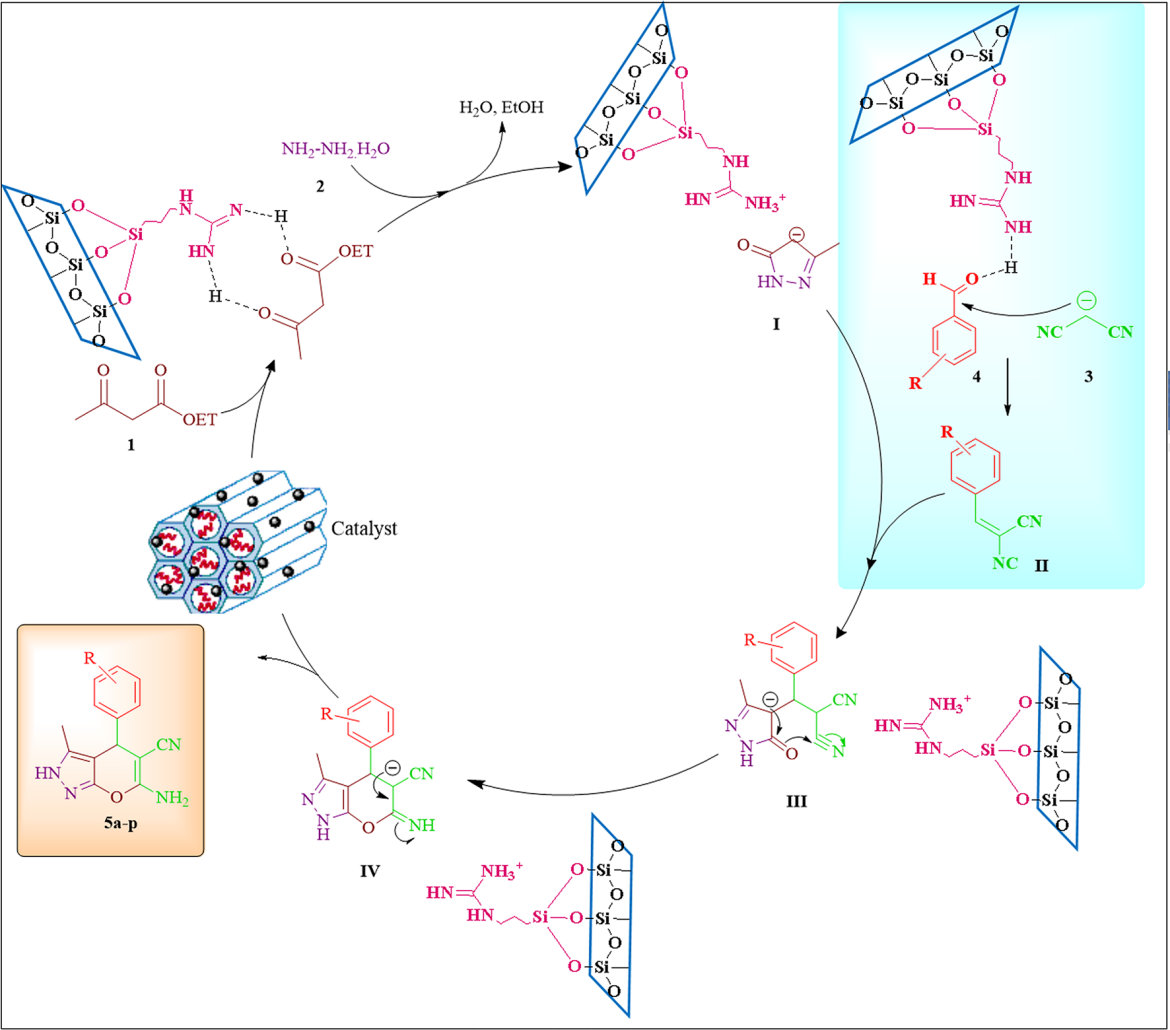
A suggested mechanism for the synthesis of pyrano[2,3-c]pyrazole derivatives catalyzed by the guanidinylated SBA-15/Fe_3_O_4_.

As mention earlier, guanidinylated SBA-15/Fe_3_O_4_ mesoporous nanocomposite with dual active sites: base (primary and secondary amines in guanidine groups), Lewis acid site (Fe^3+^ in Fe_3_O_4_), high porous structure and large surface area, play an important role in all steps of this four-component reaction as illustrated in Fig. 10. As reported in the previous studies^22,47^, several main steps give the final product. First, the carbonyl groups of ethyl acetoacetate are activated by both amine group through hydrogen bonding and the Lewis acid site in the guanidinylated SBA-15/Fe_3_O_4_ catalyst and then subjected to the nucleophilic attack of hydrazine hydrate with two nucleophilic sites. In this step, a pyrazolone ring is formed (intermediate **I**) by removing water and ethanol molecules, respectively. On the other hands, intermediate **II** (2-phenylidenemalononitrile) was produced through the Knoevenagel condensation reaction between catalysts-activated malononitrile (by strong basic sites of catalyst) and activated aromatic aldehyde (by hydrogen bond and Lewis acid site). Then, the Michael addition reaction between catalyst-activated the intermediate **I** and **II**, resulted in the compound **III**. The subsequent enolization and cyclization of intermediate **III** provided compound **IV**. Eventually, by tautomerization of molecule **IV**, dihydropyrano[2,3-c]pyrazole derivatives (**5a-p**) were obtained.

### Reusability evaluation of guanidinylated SBA-15/Fe_3_O_4_ mesoporous catalyst

**Figure 11:**
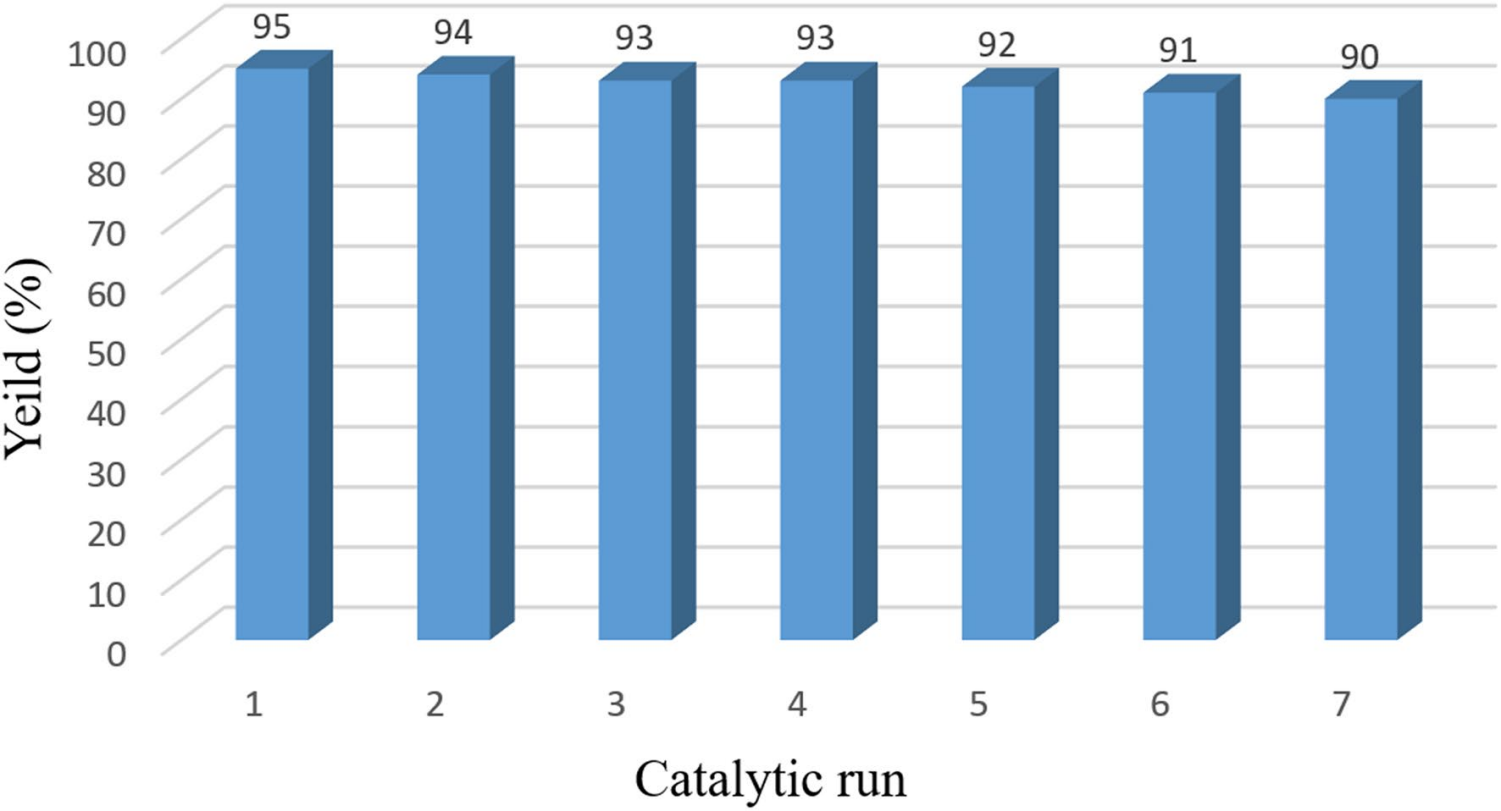
Recycling diagram of the guanidinylated SBA-15/Fe_3_O_4_ mesoporous catalyst in the synthesis of **5e**.

The recovery and reusability of catalysts in catalytic reactions are important issues in term of the green chemistry^21,28,55^. The reusability of prepared mesoporous catalysts in the synthesis of pyranopyrazoles derivatives was evaluated in several runs. For this, after completion of the reaction, the catalyst was separated from the reaction mixture by a magnet, eluted by ethanol and dried in an oven at 60 °C for 6 h in order to be ready for the next catalytic run. Later, the recovered catalyst in a constant amount was used for the subsequent runs. The presented results in Fig. 11 revealed that the recycled catalyst could be effective at least six consecutive runs without considerable reduction in its catalytic activity.

## Conclusion

A novel mesoporous SBA-15 based nanocomposite prepared by modification of SBA-15 through *in-situ* construction of Fe_3_O_4_ MNPs, consequent functionalization by APTES and finally guanidinylation reaction. This fabricated nanocomposite exhibited great catalytic performance in the synthesis of dihydropyrano pyrazole. The corresponding products were obtained in high yields without a complicated work-up procedure. The result of TGA analysis indicated that this nanocomposite has a very high thermal stability and has lost only about 15% of its weight up to 800 °C. The XRD pattern of nanocomposite in comparison with SBA-15 revealed that fabrication of Fe_3_O_4_ MNPs with crystalline nature on SBA-15 porous support, even with two consecutive chemical modifications has enhanced its crystallinity. Moreover, presented peaks in diffractogram of guanidinylated SBA-15/Fe_3_O_4_ mesoporous were in good agreement with characteristic peaks of SBA-15 and standard Fe_3_O_4._ The VSM analysis demonstrated the superparamagnetic property of nanocomposite with a Ms of about 12.2 emu/g. The FESEM images of SBA-15 and mesoporous nanocomposite showed the porous structure of SBA-15 and distribution of spherical shaped Fe_3_O_4_ MNPs on SBA-15 support with an average size of about 26 nm. The FESEM of SBA-15 and mesoporous nanocomposite showed the porous structure of SBA-15 and distribution of spherical shaped Fe_3_O_4_ MNPs on SBA-15 support with an average size of about 26 nm, and TEM images of nanocomposite exhibited both a regular mesoporous arrangement with two-dimensional hexagonal honeycomb structure and the Fe_3_O_4_ MNPs onto SBA-15 support. Based on the suggested mechanism, the guanidinylated SBA-15/Fe_3_O played a vital role in conducting the synthesis reaction of dihydropyrano[2,3-c] pyrazole derivatives with beneficial features such as strong basic sites, Lewis acid site, porous architecture and high surface area.

## Supplementary Information

Supplementary Information.
