# Supplementary material for: Guanidinylated SBA-15/Fe3O4 mesoporous nanocomposite as an efficient catalyst for the synthesis of pyranopyrazole derivatives
Source: Sci Rep. 2021 Oct 6;11:19852. doi: 10.1038/s41598-021-99120-3 (PMC8494731; doi:10.1038/s41598-021-99120-3)
Supplement: Supplementary file 1 — Supplementary Information. [file 41598_2021_99120_MOESM1_ESM.doc]

**Supporting information**

**Guanidinylated SBA-15/Fe_3_O_4_ mesoporous nanocomposite: preparation, characterization, and catalytic application in the synthesis of dihydropyrano[2,3-c]pyrazole derivatives**

Fereshte Hassanzadeh-Afruzi, Somayeh Asgharnasl, Sara Mehraeen, Zeinab Amiri-Khamakani, Ali Maleki*

Catalysts and Organic Synthesis Research Laboratory, Department of Chemistry, Iran University of Science and Technology, Tehran 16846-13114, Iran

**Corresponding author E-mail:* [*maleki@iust.ac.ir*](mailto:maleki@iust.ac.ir)*; Fax: +98-21-73021584; Tel: +98-21-73228313*

| **Table of contents** |
| --- |
| *Subject Page* |

Copies of ^1^H and ^13^C NMR spectra of compounds **5b**, **5e**, **5k** (Figure S1-S6…….……… S2-S7)


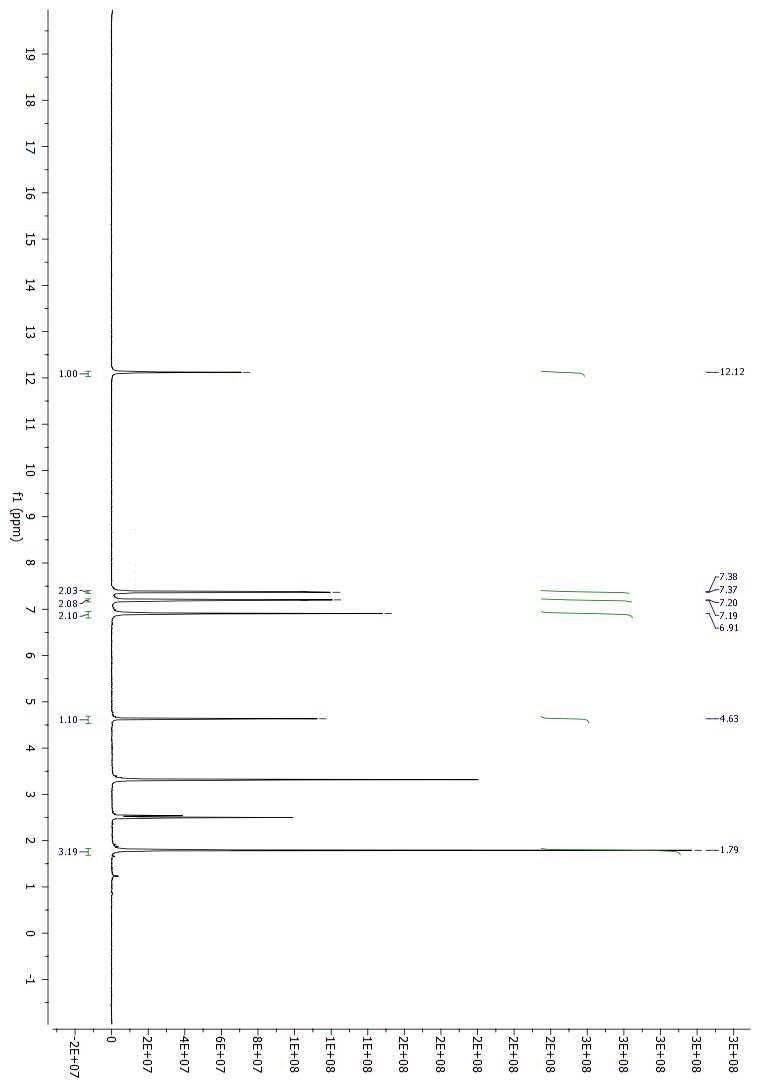


**Figure S1.** ^13^H NMR spectrum of compound (**5b**)


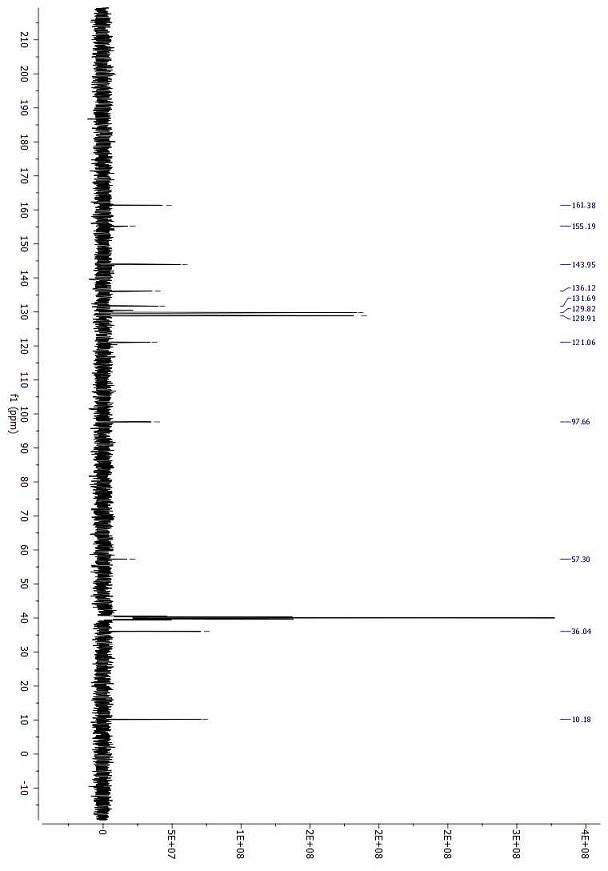


**Figure S2.** ^13^C NMR spectrum of compound (**5b**)


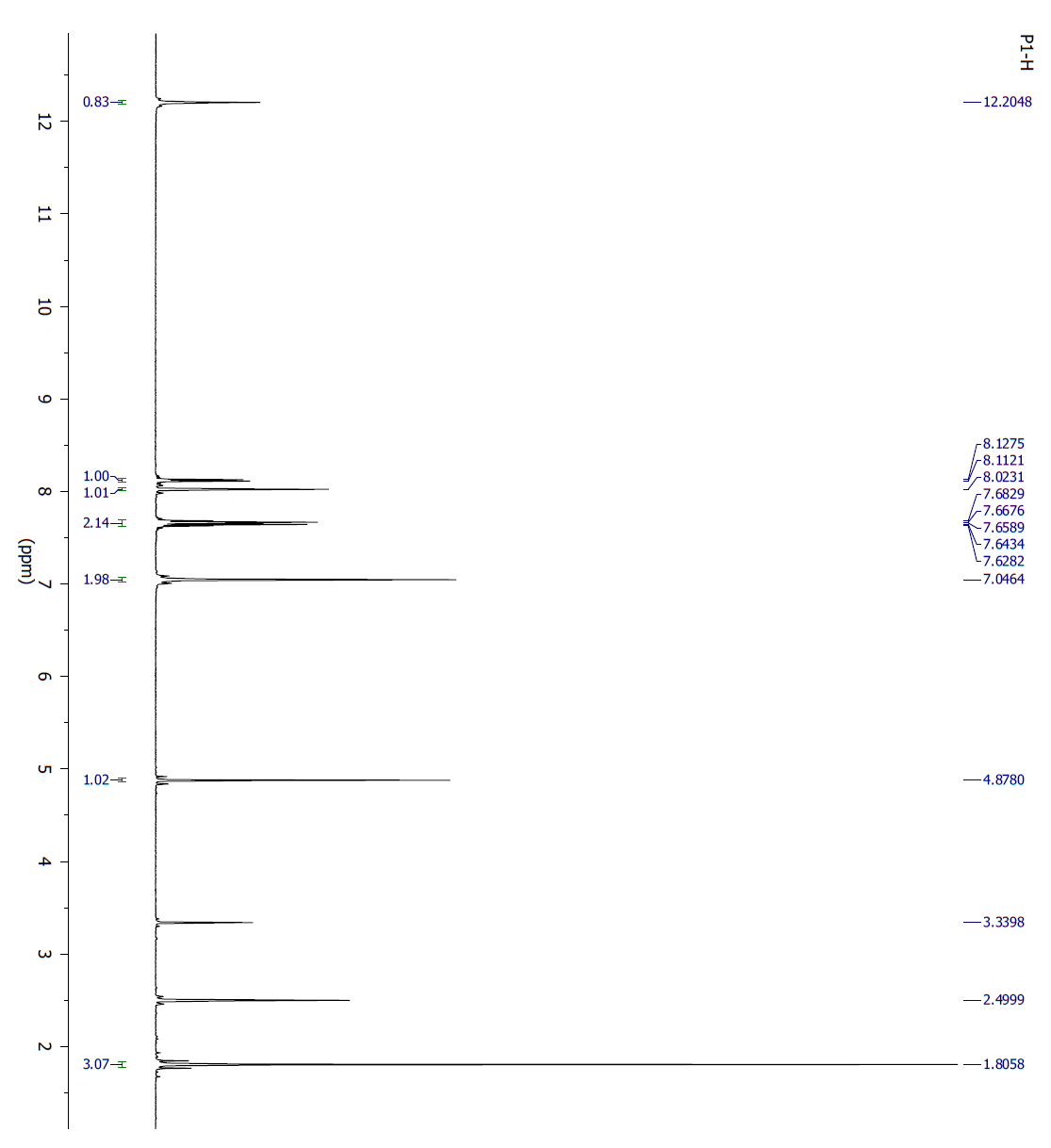


**Figure S3.** ^13^H NMR spectrum of compound (**5e**)


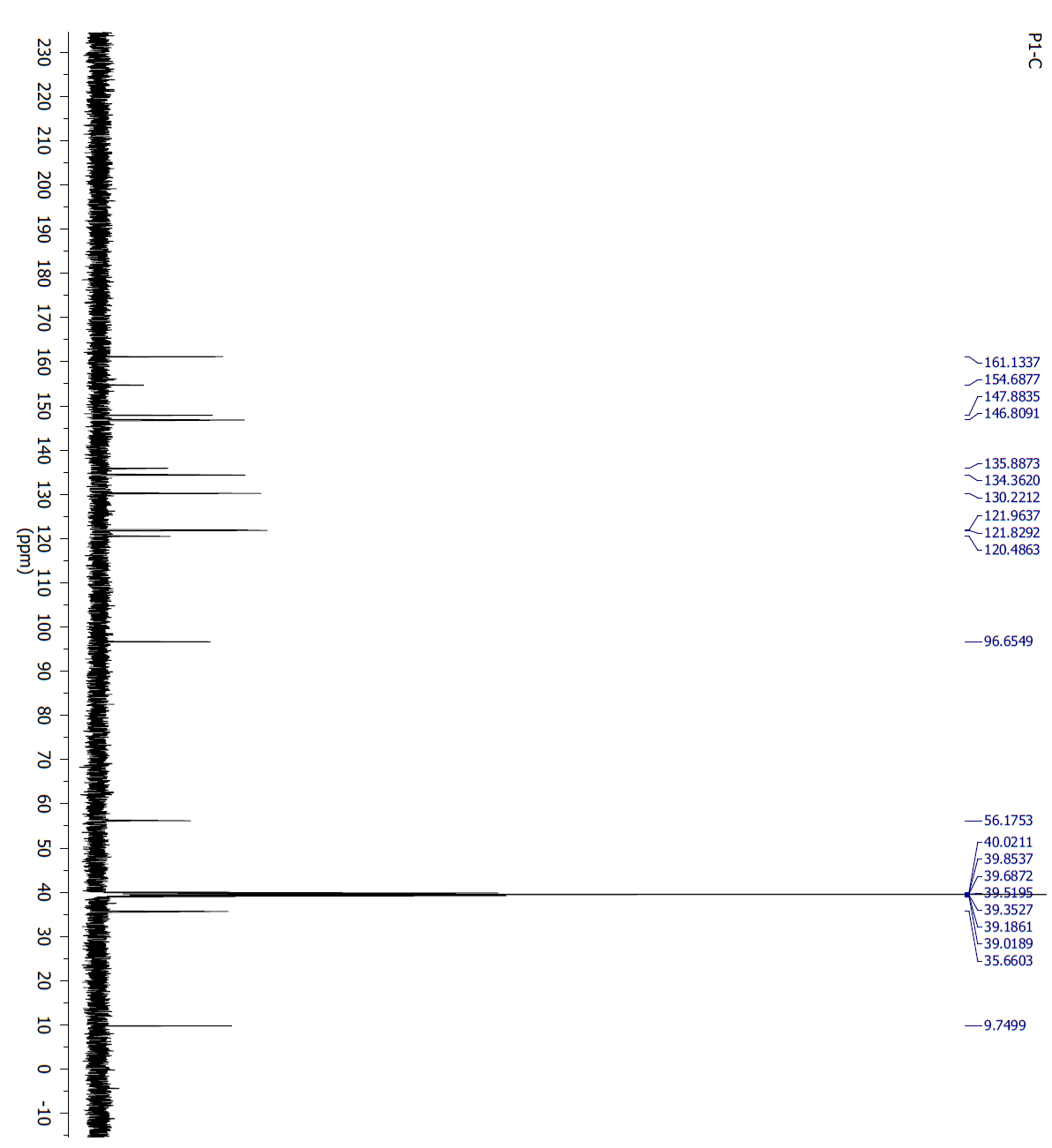


**Figure S4.** ^13^C NMR spectrum of compound (**5e**)


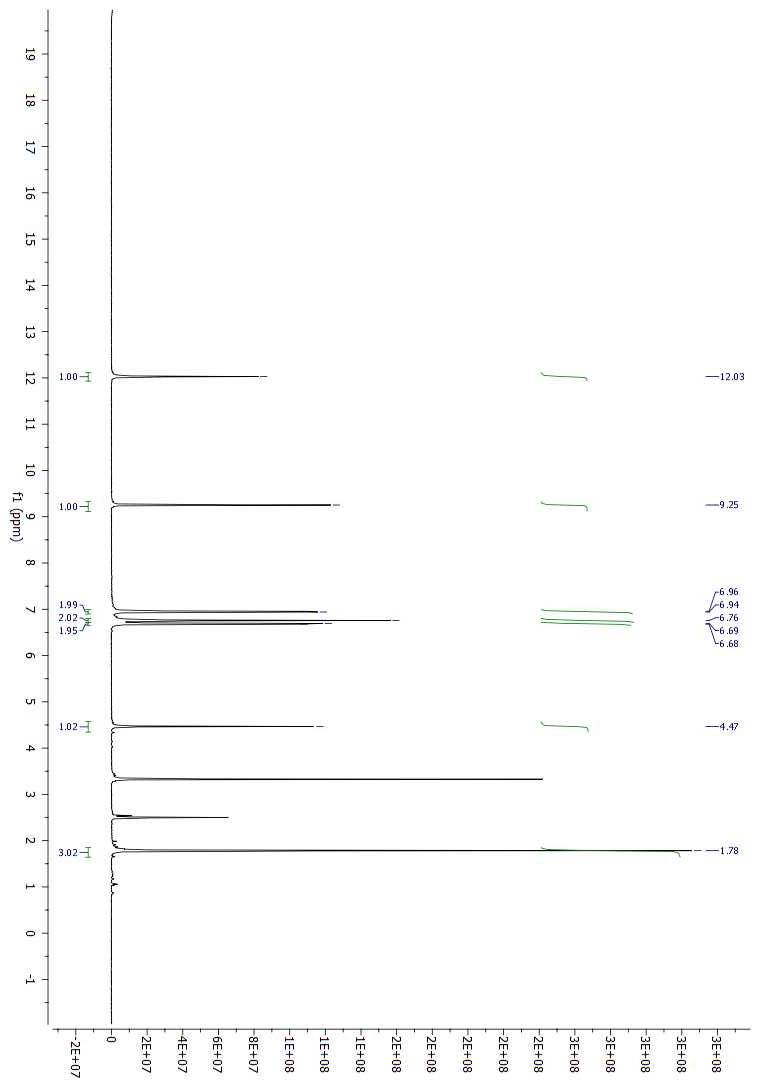


**Figure S5.** ^13^H NMR spectrum of compound (**5k**)


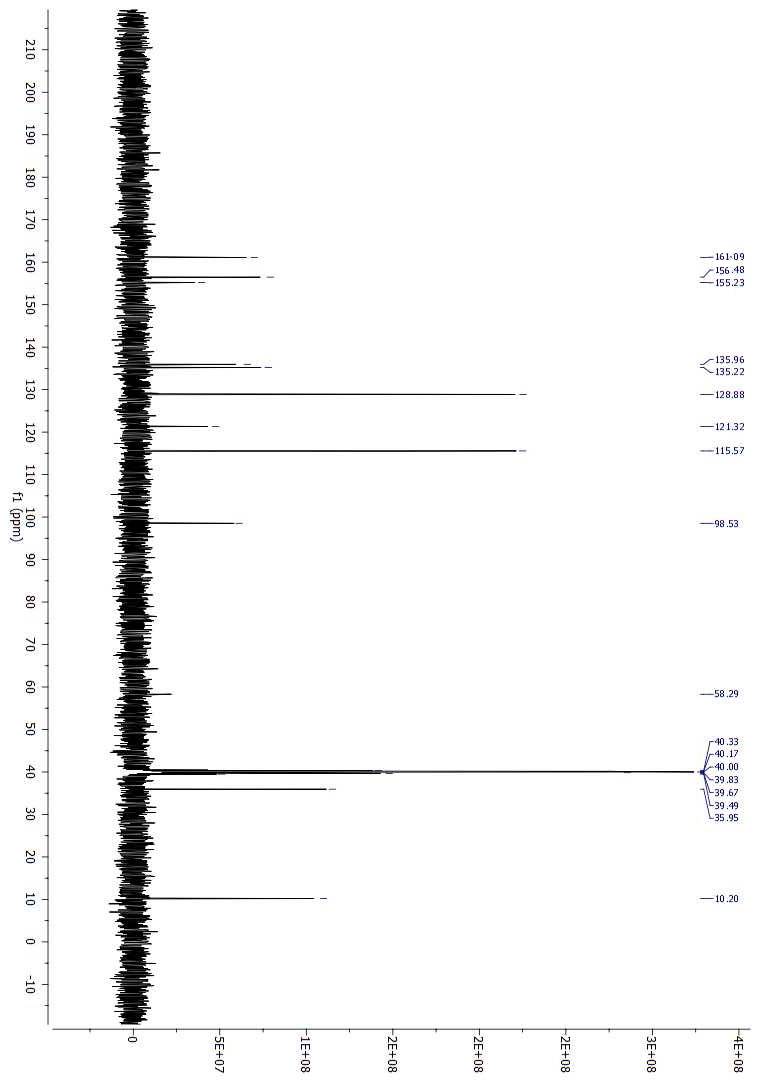


**Figure S6.** ^13^C NMR spectrum of compound (**5k**)
